# Design and Usability Study of a Point of Care mHealth App for Early Dry Eye Screening and Detection

**DOI:** 10.3390/jcm12206479

**Published:** 2023-10-12

**Authors:** Sydney Zhang, Julio Echegoyen

**Affiliations:** Department of Clinical Research, Westview Eye Institute, San Diego, CA 92129, USA; jechegoyen@wvoptometry.com

**Keywords:** dry eye disease, diagnosis, mHealth, point of care, smartphone app, eye blink, EyeScore, eye healthiness score

## Abstract

Significantly increased eye blink rate and partial blinks have been well documented in patients with dry eye disease (DED), a multifactorial eye disorder with few effective methods for clinical diagnosis. In this study, a point of care mHealth App named “EyeScore” was developed, utilizing blink rate and patterns as early clinical biomarkers for DED. EyeScore utilizes an iPhone for a 1-min in-app recording of eyelid movements. The use of facial landmarks, eye aspect ratio (EAR) and derivatives enabled a comprehensive analysis of video frames for the determination of eye blink rate and partial blink counts. Smartphone videos from ten DED patients and ten non-DED controls were analyzed to optimize EAR-based thresholds, with eye blink and partial blink results in excellent agreement with manual counts. Importantly, a clinically relevant algorithm for the calculation of “eye healthiness score” was created, which took into consideration eye blink rate, partial blink counts as well as other demographic and clinical risk factors for DED. This 10-point score can be conveniently measured anytime with non-invasive manners and successfully led to the identification of three individuals with DED conditions from ten non-DED controls. Thus, EyeScore can be validated as a valuable mHealth App for early DED screening, detection and treatment monitoring.

## 1. Introduction

The eye is one of the most important organs, and about 80% of outside information is processed by the visual pathway [1]. Human tears play a crucial role in the protection and lubrication of the ocular surface, and its secretion is mainly through the lacrimal gland [2]. The function of blinking is to bring fresh tears and nutrients over the ocular surface, while also removing metabolic waste or debris [3,4]. Research has shown that the eye blink rate normally ranges from 15~20 blinks per minute [5,6]. Dry eye disease (DED) is the most common eye disease with a prevalence ranging from 5% to 50% globally [7], which is projected to significantly increase in the coming years [8,9]. Dry eye patients usually experience blurry vision with a dry and gritty feeling in their eyes [10,11], blinking functions as a compensatory mechanism for their dysfunctional and unstable tear film. More than two times higher blink rates were widely reported in DED patients as compared to the normal controls [3,12]. In addition, partial blinks were also a common blink pattern in DED patients and featured no contact between the upper and lower eyelids. It was reported that OSDI and the number of partial blinks were positively correlated [13]. Besides blink rate and partial blink, comprehensive studies also showed that extended lid contact time, interblink interval and maximum blink interval (MBI) can be critical biomarkers for the early diagnosis of DED [12,13,14,15].

Recent development in medical big data and mHealth offers a great opportunity for DED patients to safeguard their eyecare [16]. Since early diagnosis is the key to preventing DED from progression, creating a point-of-care (POC), real-time, user-centered digital tool targeting relevant biomarkers (such as eye blink rate and partial blink rate) can effectively address the critical challenges and provide significant clinical values for personalized diagnosis with early intervention and the treatment of DED [17].

However, very few mHealth Apps are currently available for dry eye diagnosis. In Japan, efforts have been made recently to develop a mHealth smartphone App called “DryEyeRhythm” or “DEA01” for dry eye diagnosis assistance [18] (DryEyeRhythm can be downloaded from the Apple App Store with some contents in Japanese). This mobile App mainly uses the Japanese version of the OSDI questionnaire (J-OSDI) as well as App-based MBI measurement for DED diagnosis. The initial clinical assessment of DryEyeRhythm exhibited positive results with satisfactory internal data consistency [19,20]. It was reported that patient enrollment has started in Japan for a clinical trial of DEA01 in 2023 [21].

The goal of our current study is to design an iOS smartphone App called EyeScore, which can be used to accurately measure blink rate and blink patterns through advanced computational algorithms, followed by an App-based questionnaire for demographic information and eye disease symptoms or history. Then, a clinically relevant and personalized “eye healthiness score” can be reported in real-time after each test. This 10-point eye healthiness score was designed to serve as an indicator of the eye conditions of the patient, which can be non-invasively measured with the EyeScore App anytime at home and shared with the patient’s eye doctor remotely in low-resource settings. The primary purpose of the EyeScore App is to rapidly screen for patients at the early stage of DED or with mild DED conditions through the frequent in-home monitoring of eye conditions at no cost. Moreover, EyeScore can also be used to monitor eye conditions during DED treatment courses. Our initial test of EyeScore with 20 participants (including 10 confirmed DED patients) demonstrated encouraging results, with 3 of 10 non-DED participants identified with mild DED conditions. This proof-of-concept study paved the way for the EyeScore App to be further evaluated for early DED screening and detection in large-scale clinical validation studies in the future.

## 2. Materials and Methods

### 2.1. Study Participant Selection and Group Allocation

A total of 20 test participants were included in this study with age ≥ 18 years old. The written informed consent was obtained from each participant before taking a 1 min video recording. All involved parties in this study made efforts to protect study patients’ personal information and privacy. The test participants were allocated at a 1:1 ratio into the DED group (including 10 DED patients with confirmed diagnosis) and the non-DED group (10 individuals with no known eye disease conditions). Table 1 shows the demographic data of 20 participants in this study, including their gender, sex and occupation. In addition, daily electronics use time and pre-existing eye diagnosis or symptoms were also documented. A 1 min video was recorded for each participant according to the standard protocol described in the following section.

### 2.2. Video Recording Conditions and Digital Data Storage

**Figure 1:**
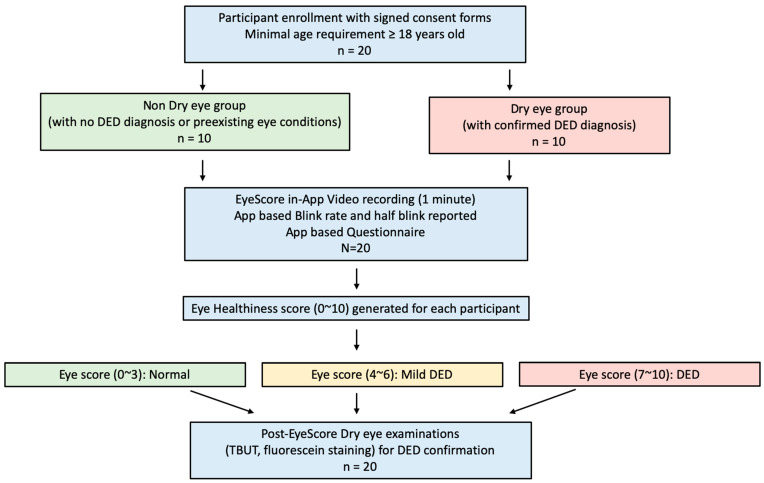
A study flow chart of EyeScore App evaluation for DED screening and detection.

Standardized iPhone video recording conditions were as follows: (1) Distance, the viewing distance from the eyes to the iPhone camera was one arm length (~76 cm or ~30 inches). An adjustable iPhone stand was used in this study, while handheld iPhone recording at arm’s length was also acceptable. (2) Lighting, normal indoor lighting with a simple background. (3) Time, 1 min recording time. (4) Resolution, HD resolution was used in our study, but 4k resolution was also acceptable for data processing. (5) Video data storage, all in-app recording videos were securely stored both in the archived database at Westview Eye Institute (San Diego, CA, USA) and the Apple iCloud private account. Figure 1 shows a study flow chart from participant enrollment to EyeScore App testing and post-EyeScore DED diagnosis confirmation.

### 2.3. Computer Programming Tools and Software Development Package

For EyeScore App development, Swift programming was performed with an Apple MacBook Pro laptop installed with iOS 16.1.2 and Xcode 14.0. The Apple Developer documentation was adopted for downloading VNFaceLandmarks2D for the development of eye landmark detection and data array acquisition (Apple Developer Documentation, 2023).

### 2.4. EAR-Based Algorithm Development for Blink Count

**Figure 2:**
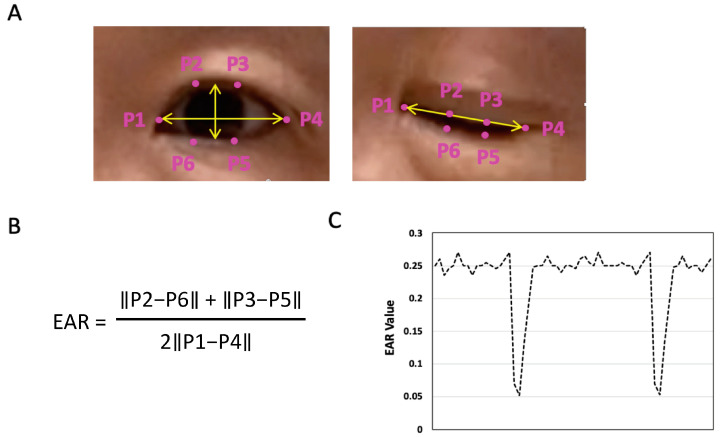
Eye landmarks (P1~P6) and EAR calculation formula for 2D eye blink detection. (**A**) The locations of six eye landmarks (P1 to P6) around the eye during the eye opening (left) or closing (right) state. (**B**) The formula for calculation of the EAR values. (**C**) The EAR values decrease when the eye is closed.

Soukupova and Cech first proposed an algorithm using six landmarks around the eye, namely P1 to P6 (Figure 2A) from 2D images [22]. A single scalar parameter called “eye aspect ratio” (EAR) was created as a determinant of the eye opening (Figure 2A, left)/closing (Figure 2A, right) state with a computational formula shown in Figure 2B. The EAR value remains relatively constant (~0.25, Figure 2C) when eyes are open, but rapidly decreases to near 0 when eyes are closed (Figure 2C). As a result, the successful use of a fixed-value EAR threshold led to the development of eye blink counts for a 3D video stream with OpenCV, Python, and Dlib [23,24,25].

The development of eye landmark recognition and EAR thresholds made it possible to use a similar approach in the Xcode-integrated environment for an iOS App development. The authors in the current study proposed to use a 1-min iPhone video so the reliable eye blink behavior of each participant can be captured through in-app recording and subsequently stored in the iPhone photo library. For video processing, the EAR formula from Figure 2B was utilized initially to calculate the EAR value of each video frame, with the reporting of the individual EAR, Average EAR, MaxEAR, and MinEAR from all processed frames.

**Figure 3:**
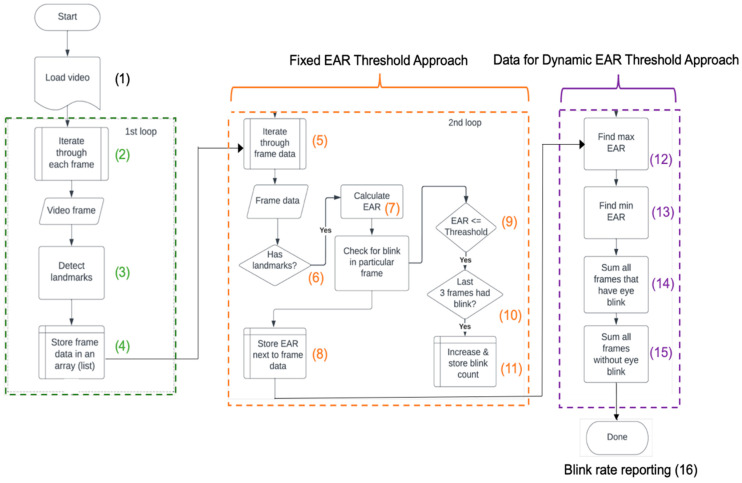
Programming flow chart with fixed EAR approach with the obtained data for dynamic EAR approach. Three loops of code blocks were labeled with green, orange, and purple colors, with (1) to (16) referring to the numbered step in the diagram.

Figure 3 shows the schematic programming flowchart of the EyeScore App, which includes three code blocks labeled with green, orange, and purple colors. The detailed programming procedures with the numbered steps (1)–(16) were described in Supplemental Materials S1.

However, a fixed-value threshold approach failed to correctly report the blink rate and partial blink, particularly when tests were performed with dry eye patients. This was because DED patients frequently show abnormally high partial blink rates. Using data obtained from the purple block (Figure 3), a dynamic EAR-value-based algorithm was developed as a personalized threshold to determine the eye opened or closed state for this study.

### 2.5. Creation of an Eye Healthiness Score for Monitoring Eye Conditions

Based on the literature review on DED, a 10-point formula was proposed in this study with five components for computing an eye healthiness score (Table 2). Two components (blink rate and partial blink rate) were reported from video-based analysis with EyeScore App, while the other three components were obtained from the questionnaire filled out by the study participants. These three components were the most common risk factors known for DED development [26,27]. The supporting references for each of the components are also included in Table 2.

Briefly, (1) Blink rate. The normal blink rate ranges from 15–20 per minute, while a blink rate over 30 per minute is considered a high probability of DED [5]. It carries a three-point weight in our formula. (2) Partial blink. The partial blinks are common in DED patients due to their dry and dysfunctional ocular surface, while normal controls should have partial blinks < 5 per minute [12,13]. It also carries 3 points in the formula. (3) Dry and gritty feeling. clinically called “foreign body sensation” [26]. The patients start to develop DED symptoms at this stage so it carries two points in the formula. (4) Women over 50 years old. This group of women shows a statistically higher risk of DED development among all population groups [26]. It carries 1 point in the formula. (5) Regular contact lens wearers. Many publications show that regularly wearing soft or hard contact lens have a significantly higher risk of inducing DED [27]. Thus, a regular contact lens wearer (not occasional use of contact lens) carries one point in the formula.

Although many other risk factors also contribute to the proposed eye healthiness score for DED diagnosis [28], our initial formula focused on the most important factors, with a score ≥ 4 being considered as mild DED conditions. When the eye healthiness score ≥ 7, the EyeScore App will report it as severe DED and recommend the user schedule doctor’s visit to confirm the DED diagnosis (Table 2). A healthy individual with normal eye conditions should have a score ≤ 3. Although the weight/points for this formula have significant limitations, the authors decided to use this scoring system for our small pilot study. The proposed eye healthiness score can be further optimized in the future with other important risk factors [28] for linear regression analysis [29].

### 2.6. Statistical Analysis

The student *t*-test was performed using GraphPad Prism 9.0 (GraphPad Software Inc, San Diego, CA, USA). The statistical significance was determined by *p*-values as follows: *p*-values of <0.05; <0.01; and <0.001. The mean ± standard deviation was obtained from at least three separate experiments.

## 3. Results

### 3.1. EyeScore App Interface and Questionnaire

**Figure 4:**
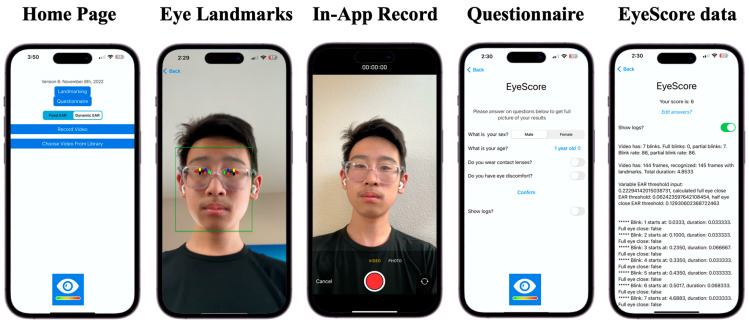
Representative EyeScore App functions and user interfaces.

Selected EyeScore App functions and user interfaces are shown in Figure 4, which include a home page, eye landmark detection, in-app recording, questionnaire, and eye healthiness score report. The patient registration and account information will be available in the future so the personalized EyeScore results can be securely stored and shared with eye doctors for remote monitoring of eye conditions.

### 3.2. Limitations of a Fixed Value EAR Approach

For EyeScore App algorithm development, videos from six controls and four DED patients were used for the initial development of a fixed-value EAR (Table 3). To study the effects of masks on the blink count, two individuals (Enrollee 3 and 6) in the control group recorded the EyeScore videos without masks (3A, 6A) and with masks (3B, 6B). An initial program code was written to allow the EyeScore App to iterate through all video frames for eye landmarks., followed by the calculation of EAR for each frame. If there are three or more frames in a row with an EAR value ≤ a fixed-value EAR threshold, the App counts this sequence of frames as a single blink. The blink rate can be reported after the combination of all validated blinks in one minute. As seen in Table 3, a fixed value EAR of 0.163 was suitable for classifying open and closed eye states for three study participants in the six-member control group (video no. 1~6 in Table 3). For example, for videos 1, 2 and 4, blink count results from the EyeScore App with fixed value EAR vs. manual count was 100% matched (Table 3). However, for all 4 confirmed DED patients (Video No. DED1~DED4) and three controls (Video No. 3A, 3B, 5, 6A, 6B), the blink count results were significantly lower due to the partial blinks not being counted (Table 3). As shown in Figure 2B, the EAR value was based on the distance between eye landmarks on the upper and lower eyelids. The EAR values of partial blinks were significantly increased due to the unclosed, separated eyelids, thus preventing them from being counted. Another variable was the mask. participants wearing a mask can significantly interfere with landmark recognition from VNFaceLandmarks2D, with many frames returned with “no landmark identified”. Video 3B and 6B represented examples of mask-wearing with significantly low blink counts as compared to the manual counts. Thus, the authors decided to use videos with no masks for the rest of the study. Moreover, wearing frame glasses was not allowed when taking the video recordings due to the various refractive indices of the lens.

In addition to the partial blink, different study participants (particularly DED patients) exhibited a range of eyelid patterns when in the eye fully opened state or in the eye fully closed state. As a result, the MaxEAR value and the MinEAR value can be significantly different. The use of a single fixed value EAR as a threshold led to inaccurate results of full blink and partial blink counts, as shown in Table 3. Thus, a dynamic, individualized EAR threshold that considers these variables was essential to address these challenges for improving the accuracy of full blink and partial blink counts, particularly in DED patients.

### 3.3. Development of a Dynamic EAR Approach with Two EAR Thresholds

Efforts were made to develop algorithms to two separate counts for full blink and partial blink, respectively. Two EAR thresholds were essential for EyeScore to process the 3D videos for reporting separated full blink and partial blink rates. Of these two EAR thresholds, one was defined as the full blink EAR threshold, while the other was called the partial blink EAR threshold. After multiple tests of coefficient calculation for accurate blink counts, the following algorithm was chosen to calculate the full blink EAR threshold value:

Full blink EAR threshold = (0.1281/0.27527) × (MaxEAR − MinEAR)

The MaxEAR and MinEAR values were obtained from steps (12) and (13) in Figure 3.

Meanwhile, the partial blink EAR threshold value was also defined as:

Partial blink EAR threshold = Full blink EAR threshold value × 1.4

In other words, a partial blink occurs when the frame EAR value is 40% higher than the full blink EAR threshold. The two resulting dynamic threshold values were designed to be more stringent and tied to the MaxEAR and MinEAR of the testing individual, which allowed for the accurate reporting of full blink and partial blink count from all 20 recorded videos (Table 4).

### 3.4. Accurate Blink and Partial Blink Results Obtained from the Dynamic EAR Approach

As shown in Table 4, a total of 20 videos from 10 confirmed DED (including four DED patients under clinical treatments) and 10 normal controls (no DED diagnosis or symptoms) were analyzed with the newly established dynamic EAR thresholds for full blink and partial blink counts. The full blink counts from 20 videos were 100% matched with manual counts, validating our dynamic EAR thresholds approach. Importantly, EyeScore can accurately count partial blinks with the newly developed partial blink EAR threshold, which is much more precise and efficient than the manual count. The accurate measurements of both full blink and partial blink lay a solid foundation for reporting App-based eye healthiness scores. The average full blink rate for control and DED groups were 22.2 and 37.4 blinks per minute, respectively, while the average partial blink rate for control and DED groups were 2.5 and 5.8 blinks per minute, respectively (Table 4). Although the partial blink rate did not show significance statistically with a *p* value > 0.05, the three “mild DED” patients with high partial blink rates in the control group technically interfered with the results of Student’s *t*-test (Table 4). Once these three patients were moved to the DED group, the partial blink rate became statistically significant (*p* < 0.05) between the control and the DED groups.

The results from the EyeScore report confirmed all 10 known DED patients, with four of them with scores ranging from 7 to 9 (severe DED category), while six of them with scores ranging from 4 to 6 (Mild DED category). Interestingly, all four DED patients with a high score of 7–9 showed longer daily electronic use time (DED 4~6, DED10 in Table 1). On the other hand, four of six DED patients scoring 4~6 were undergoing clinical DED treatment. Thus, the relatively lower eye healthiness score could be the treatment results from the improved eye conditions. In comparison, 7 of 10 normal controls exhibited eye healthiness scores from 0 to 3, confirming their normal eye healthiness conditions. Importantly, 3 of 10 normal controls (i.e., patients No. 5, 6 and 7, with no previous DED diagnosis from their eye doctors) demonstrated mild DED conditions. They either exhibited significantly higher partial blink rates (in enrollees 5 and 6) or higher full blink rates (enrollee 7) compared to other healthy individuals. As a result, their eye healthiness scores were reported as 4, 6, and 5, respectively. Importantly, our post-EyeScore eye examinations showed that these three participants had a shortened TBUT time (<10 s) and positive fluorescent staining, confirming they were in the early phase of DED development (Table 4). For patients with scores ≥ 4, the EyeScore App was designed to send an alert notification of mild DED conditions to the user. These patients were recommended to change their daily eye usage routines with healthy habits, such as taking breaks from extended computer use and increasing outdoor activities.

## 4. Discussion

The multifactorial nature of DED pathogenesis has made its diagnosis and treatment a significant challenge among common eye diseases [10,30]. Current DED diagnosis mainly relies on clinical tests such as TBUT, OSDI, Schirmer’s test, and fluorescein staining, which can only be performed in an eye doctor’s office [31]. This can significantly delay the early diagnosis of DED, which often leads to irreversible chronic DED conditions. As a result, a great number of DED patients remain undiagnosed and inadequately treated, particularly in our ever-expanding digitalized society. Thus, a POC, mHealth diagnosis tool can bridge the gap and offer digital, low-cost, patient-centered solutions to the millions of under-diagnosed DED patients [16,17]. Although the smartphone App DryEyeRhythm was developed and tested for DED diagnostic assistance in Japan [18], our EyeScore App aims to perform rapid, accurate, in-home DED diagnostic screening, detection, and treatment monitoring. Most importantly, the eye healthiness score was uniquely created as a convenient digital parameter for the real-time reporting of DED eye conditions. EyeScore mHealth App can benefit both DED patients and their eye doctors with the following features.

First, eye blink and partial blink rates provide reliable biomarkers for early DED diagnosis [12,13]. Comprehensive studies on eye blink patterns strongly support their specific roles in the DED development process, either as a compensation mechanism for supplying more tears to the inflammatory ocular surface or as resulting symptoms from dry eye development. Thus, the proposed formula for eye healthiness score weighs heavily on both full blink rate and partial blink, with a total of 6 points on the 10-point scale. The unique approach for separated calculation of thresholds for full blink and partial blink further ensures the accuracy of blink counts, which serves as the foundation for early DED screening through the EyeScore mobile App. Our study strongly supports our eye blink counts approach, with 8 of 10 known DED patients exhibiting >30 full blinks per minute. In addition, five DED patients also demonstrated >5 partial blinks per minute. As a result, all participating DED patients showed their eye healthiness score in the range of 4~9 (Table 4), confirming that the EyeScore App and the eye healthiness score are excellent digital tools for DED rapid screening and detection.

Second, the eye healthiness score was carefully designed to cover common causes or symptoms of the multifactorial DED [26], with the inclusion of dry/gritty feelings (foreign body sensation), gender/age and regular contact lens wearing [27]. Since our focus was to perform App-based screening and early dry eye detection, the initial questionnaire was designed to obtain objective information from the EyeScore users, such as age, gender and eye conditions. Together with the accurate measurements of eye blink rate and partial blinks, the combination of these factors (Table 2) can give an objective, clinically relevant evaluation of eye conditions for rapid DED evaluation at home with low resource settings.

Third, the EyeScore App can be used to screen for patients with mild DED conditions at the early stage of DED development. The fact that 3 of 10 individuals in our “normal” group being identified having, with the post-EyeScore as confirmation, mild DED conditions strongly suggests that DED is significantly under-diagnosed, with a large number of hidden pre-DED and DED patients in the general population. Interestingly, the three mild DED patients identified from our “normal group” were all females with their ages ranging from 47 to 56 years old (Table 4). This subcategory of the population is well known with the highest prevalence of DED development [26]. Moreover, the use of electronics plays a significant role in DED development [32]. More than 60% of DED patients in our study have daily electronic screen time of 6 to >8 h (Table 1). With the widespread use of electronic devices in our digitalized society, a POC, mobile diagnostic method such as the EyeScore App is essential for early DED screening and prevention [17].

Lastly, the repeated in-home EyeScore exams over time can provide eye doctors with the useful trend of individual eye healthiness conditions, thus facilitating timely DED screening and detection at minimal cost. This EyeScore feature makes it possible for DED patients to set routine tests with the real-time reporting of their eye conditions, similar to other health parameters, such as BMI, heart rate and blood pressure. For DED patients undergoing treatment (such as artificial tears, Omega-3 and warm compress), EyeScore also allows doctors to remotely monitor DED treatment courses and make necessary changes of regimens accordingly. In our study, four of ten DED enrollees were under various forms of DED treatment (Table 4). All of them demonstrated decreased eye healthiness scores over a 2-to-4-week period after treatment was initiated (internal data). Thus, the EyeScore App can improve the prognosis of these DED patients without the need to visit eye doctors’ offices in person. In addition, the EyeScore mobile App can feasibly collect a large amount of DED patient data that was previously inaccessible through traditional clinical methods. Encouraged by these positive results, the advanced development of the EyeScore App is also planned for this mHealth project, with possible machine learning algorithms and statistical analysis to further optimize the accuracy of blink count and eye healthiness score, with a particular focus on the identification of patients with pre-DED conditions.

Although the EyeScore App effectively identified all DED patients as well as three patients with mild DED conditions from the control group, caution should be taken for the data interpretation with our small sample size pilot study. Given the multifactorial nature of DED pathogenesis and only three factors being considered for the current study, our EyeScore results exhibited certain limitations and biases. In many cases, the DED patients were under different clinical treatment methods, further complicating the data interpretation. One possible solution is to perform a large trial study with three groups, including DED untreated, DED treated, and controls. In addition, many other conditions (such as smoking, alcohol, refractive surgery, glaucoma and autoimmune diseases) were known to be important factors in DED pathogenesis [28]. These factors should be included in eye health score calculations in the future when enough patient data from relevant clinical subgroups becomes available with statistical significance.

The smartphone-based platform offers great clinical potential for future POC diagnostic methods [33]. As the patient data collection for digital recording grows along with integrated user inputs, the eye blinking recognition signals can be further analyzed, quantitated, and categorized for fine-tuning the proposed algorithms [34]. Further machine learning and artificial intelligence-based modeling can provide even deeper, hidden connections of eye blinking patterns of DED or other ocular diseases [35,36]. Such a data-driven approach will ultimately lead to the establishment of DED diagnosis toward predictive, preventive, personalized and participatory medicine [37].

## Figures and Tables

**Table 1:** Demographic data from 20 participants in this study.

Group	Enrollee No.	Gender	Age	Occupation	Electronics Use ^†^	Regular Contact Lens Wearing	Preexisting Eye Symptoms or Diagnosis/Treatment ^‡^
Normal	1	M	58	Researcher	+++	No	Normal
	2	F	20	Student	+++	Yes	Normal
	3	F	21	Student	+++	No	Normal
	4	F	22	Student	+++	No	Normal
	5	F	53	Homemaker	+	No	Normal
	6	F	47	Office worker	+++	Yes	Normal
	7	F	56	Homemaker	+	Yes	Normal
	8	F	80	Homemaker	+	No	Normal
	9	M	65	Cook	+	No	Normal
	10	M	29	Engineer	++++	No	Normal
DED	DED 1	F	48	Engineer	++++	Yes	Confirmed DED
	DED 2	M	80	Clinician	++	No	Confirmed DED/under treatment
	DED 3	F	83	Homemaker	+	No	Confirmed DED/under treatment
	DED4	F	21	Student	+++	Yes	Confirmed DED
	DED 5	M	60	Financial planner	+++	Yes	Confirmed DED
	DED 6	F	51	Researcher	+++	Yes	Confirmed DED
	DED 7	F	28	Clinician	++	Yes	Confirmed DED/under treatment
	DED 8	F	44	Homemaker	+	Yes	Confirmed DED/under treatment
	DED 9	M	60	Engineer	++++	No	Confirmed DED
	DED 10	M	19	Game Designer	++++	Yes	Confirmed DED

^†^ Electronic use daily (hours): +, 0~3 h; ++, 3~6 h; +++, 6~8 h; ++++, >8 h. ^‡^ All enrollees in the DED group have confirmed DED diagnosis from their eye doctors. Some of them were under DED treatment including artificial tears, omega-3, warm eye compress and blinking exercises.

**Table 2:** Different factors for computing an eye healthiness score by EyeScore App.

Factor	Point *	References
Blink rate > 30 per min	3	[5]
Partial blink > 5 per min	3	[12,13]
Dry and gritty feeling	2	[26]
Women over 50 years old	1	[26]
Regular contact lens wearing	1	[27]
Total	10	
**Normal**: 0–3; **Mild DED**: 4–6; DED: 7–10; **Severe DED** (will need a doctor’s visit)		

* Point can be optimized with other factors [28] and statistical approaches [29].

**Table 3:** EyeScore App count vs. manual count results using a fixed value EAR as a threshold.

Group	Enrollee No.	EyeScore Data with Fixed EAR	Manual	Partial Blink	Note
Controls	1	10	10	yes	Correct full blink count/no partial blink count
	2	29	29	No	Correct full blink count
	3A (no mask)	29	30	Yes	Incorrect full blink count/no partial blink count
	3B (with mask)	20	30	Yes	Incorrect full blink count/no partial blink count
	4	15	15	Yes	Correct full blink count/no partial blink count
	5	20	25	Yes	Incorrect full blink count/no partial blink count
	6A (no mask)	22	29	Yes	Incorrect full blink count/no partial blink count
	6B (with mask)	17	29	Yes	Incorrect full blink count/no partial blink count
DED	DED1	29	39	Yes	Incorrect full blink count/no partial blink count
	DED2	26	16	Yes	Incorrect full blink count/no partial blink count
	DED3	28	35	Yes	Incorrect full blink count/no partial blink count
	DED4	25	36	Yes	Incorrect full blink count/no partial blink count

**Table 4:** EyeScore App results of 20 study participants with reporting of eye healthiness scores *.

Group	Enrollee Number	Age	Gender	Preexisting Diagnosis/ Treatment	EyeScore Diagnosis	Eye Score	Full Blink Rate	Partial Blink Rate	Video Time (sec)	Open/Close Ratio (%)	Post-EyeScore Confirmation
Control	1	58	M	Normal	Normal	0	10	1	65	42.3	
	2	20	F	Normal	Normal	1	29	0	63	7.8	
	3	21	F	Normal	Normal	0	25	5	61	9.1	
	4	22	F	Normal	Normal	0	15	1	61	14.3	
	5	53	F	Normal	Mild DED	4	23	8	62	3.7	DED Confirmed
	6	47	F	Normal	Mild DED	6	21	6	62	2.4	DED Confirmed
	7	56	F	Normal	Mild DED	5	31	3	60	7.3	DED Confirmed
	8	80	F	Normal	Normal	3	22	1	60	4.0	
	9	65	M	Normal	Normal	2	28	0	62	7.9	
	10	29	M	Normal	Normal	0	18	0	58	18.4	
	Average	45	7F; 3M		Average	2.1	22.2	2.5	61.4	11.7	
DED	DED1	48	F	Mild DED	Mild DED	4	39	1	62	2.6	DED Confirmed
	DED2	80	M	DED/Treated	Mild DED	5	16	14	61	60	DED Confirmed
	DED3	83	F	DED/Treated	Mild DED	6	35	3	62	6.8	DED Confirmed
	DED4	21	F	DED	DED	7	36	8	61	3.4	DED Confirmed
	DED5	60	M	DED	DED	7	40	14	61	19.3	DED Confirmed
	DED6	51	F	DED	DED	7	64	0	61	3.0	DED Confirmed
	DED7	28	F	DED/Treated	Mild DED	6	13	6	61	9.9	DED Confirmed
	DED8	44	F	DED/Treated	Mild DED	4	23	1	61	9.8	DED Confirmed
	DED9	60	M	Mild DED	Mild DED	6	55	0	61	3.9	DED Confirmed
	DED10	19	M	DED	DED	9	53	11	61	7.2	DED Confirmed
	Average	49	6F; 4M		Average	6.1	37.4	5.8	61.2	12.6	

* (A) Enrollees with results highlighted in red color indicate DED diagnosis with abnormal eye blink rates, abnormal partial blink rates or abnormal eye scores. (B) Enrollees with highlighted yellow color results indicated newly identified DED patients from the control group with abnormal eye scores, full blink rates or partial blink rates (C) Statistical analysis with student *t*-test for data comparison between control and DED groups. (1). Full blink rate and eye score both showed *p* < 0.001 with statistical significance. (2). Partial blink and age both showed *p* > 0.05 with no statistical significance. (3). When moving three “mild DED” patients from the control group to the DED group, both full and partial blink became statistically significant (*p* < 0.05).

## Data Availability

The data presented in this study are available on request from the corresponding author. The recorded videos are not publicly available due to privacy restrictions of study participants.

## Supplementary Materials

The following supporting information can be downloaded at: https://www.mdpi.com/article/10.3390/jcm12206479/s1, The source code of EyeScore can be provided through GitHub. S1 showed the detailed step-by-step programming procedures of the EAR approach of the EyeScore App.

## Abbreviations

DED, dry eye disease; EAR, eye aspect ratio; iOS, iPhone operating system; MaxEAR, maximal EAR value; mHealth, mobile health; MinEAR, minimal EAR value; POC, point of care; TBUT, tear break up time; OSDI, ocular surface disease index.
