# Supplementary material for: Design and Usability Study of a Point of Care mHealth App for Early Dry Eye Screening and Detection
Source: J Clin Med. 2023 Oct 12;12(20):6479. doi: 10.3390/jcm12206479 (PMC10607458; doi:10.3390/jcm12206479)
Supplement: Supplementary file 1 [file jcm-12-06479-s001.zip › jcm-2627885-supplementary.pdf]

S1. The programming procedures of the EAR Approach of the EyeScore App were as follows with (1) ~ (16) referring to the numbered step in the diagram (Figure 3)

A) Green block: The video was loaded **(1)** with each frame being iterated **(2)** for eye landmark detection **(3)** via VNFaceLandmarks2D from the Apple Vision SDK package. The frame data including coordinates, starting time, and duration will be stored in an array **(4)**;

B) Orange block: The program started with iterating through all the frame data **(5)**. For each frame data with confirmed landmarks **(6)**, The EAR value will be computed using the formula in **Figure 2B (7)** with the data stored next to the frame data from the first loop in the green block **(8)**. A fixed threshold can be established as a classifier for blink count, similar to the previous publication [22]. If the EAR value is less or equal to the threshold **(9)**, the program will check whether an eye blink is observed in the last three frames **(10)**. $N \pm 3$ consecutive frames can be sufficient when their EAR values are below the blink threshold for an HD iPhone video at 30 fps (or 0.03 sec/frame) [22] on blink detection. If yes, the program increments the total number of blinks **(11)**.

C) Purple block: After two loops of code blocks (green and orange), the last set of programming is to find MaxEAR **(12)** and MinEAR **(13)** and compute all frames that have eye blink **(14)** or without eye blink **(15)**. A final blink rate will be summarized and reported **(16).**

[22] Soukupova, T.; Cech, J. Real-Time Eye Blink Detection using Facial Landmarks. In Proceedings of the 21st Computer Vision Winter Workshop, Rimske Toplice, Slovenia, 3–5 February 2016.
